# Surgical Management of Cholecystoenteric Fistula in Patients With and Without Gallstone Ileus: An Experience of 29 Cases

**DOI:** 10.3389/fsurg.2022.950292

**Published:** 2022-07-08

**Authors:** Shi-fei Huang, Ye-hong Han, Jie Chen, Jun Zhang, Hai Huang

**Affiliations:** Department of General Surgery, Hangzhou TCM Hospital affiliated to Zhejiang Chinese Medical University, Hangzhou, China

**Keywords:** cholecystoenteric fistula, cholelithiasis, complication, gallstone ileus, surgical management

## Abstract

**Background:**

Cholecystoenteric fistula (CEF) is an uncommon complication of cholelithiasis. Here, we report our experience on diagnostic methods and surgical management of CEF patients with and without gallstone ileus (GI).

**Methods:**

This is a retrospective cases series over an 11-year period (2011–2022). Data analyzed included preoperative characteristics, ultrasound, imaging features, operation findings and postoperative course.

**Results:**

A total of 29 patients diagnosed with CEF were enrolled, 51.7% (15/29) of whom were female, with a median age of 66 years (range: 35–96 years). With regards to subtype distribution, seventeen patients had cholecystoduodenal fistula (CDF), six had cholecystoconlonic fistula (CCF), three exhibited cholecystogastric fistula (CGF), one CDF combination with CCF and two CDF combination with type I Mirizzi syndrome. Twelve patients presented with gallstone ileus, and received one stage procedure or simple Enterolithotomy. The median operation time and blood loss of 157 min (range: 65–360 min) and 40 ml (range: 10–450 ml), respectively. Surgical complications, evidenced by fistula recurrence, were recorded in three patients (3/22; 13.6%), while four (4/29; 13.8%) and one patient (1/29; 3.4%) presented with wound infection and residual stone in common bile duct, respectively. No deaths were reported in our study.

**Conclusion:**

CEF is a rare complication of gallstone disease that is occasionally found during operation. To date, no consensus has been reached regarding efficacious treatment therapies for CEF patients. For a CEF patient with GI, one stage procedure should be selected prudently, while simple Enterolithotomy would be a mainstream choice for relieving bowel obstruction.

## Introduction

Cholecystoenteric fistula (CEF), a relatively rare complication of gallstone disease, is defined as an autonomous communication between the inflamed gallbladder and the surrounding adherent gastrointestinal tract (1). Previous studies have shown that the incidence of CEF ranges between 3%–5% in patients with cholelithiasis and 0.15%–4.8% in all the biliary operations (2, 3). It usually occurs at the advanced age, and most frequently presents in the duodenum where it compromises 75%–80% of all the CEF, followed by colon and stomach (4, 5). In general, GI which means a gallstone impaction within the intestinal tract, is always accompanied by CEF. The condition, first described by Thomas Bartholin in 1,654, is responsible for 1%–4% of all the mechanical bowel obstruction (1, 6). The precise preoperative diagnosis can be quite difficult, owing to a lack of specific clinical manifestations and physical signs between CEF and the uncomplicated cholelithiasis. Fortunately, advancement of imaging-diagnosis coupled with development of endoscopic techniques, have markedly improved preoperative diagnostic accuracy (4).

In this study, we retrospectively analyzed all CEF patients with and without GI, who were treated at our institution. Particularly, we assessed their diagnosis, surgical management and treatment outcomes, with the aim of generating insights to help surgeons resolve the rare problem based on the experience of our therapy center.

## Methods

The study protocol was approved by the ethnic committee of TCM Hospital affiliated to Zhejiang Chinese Medical University (No.2022KY070). We conducted an ICD-10 code retrieval (code K82.301, K82.302, K82.303, K82.304) in our clinical database from Jan 2010 to Dec 2021 to identify patients with CEF. Overall, we enrolled 29 patients with CEF (12 patients with GI), while those treated conservatively without surgical intervention were excluded. We collected and analyzed their demographic data, clinical manifestations, physical signs, laboratory test, medical examinations, operation dates, postoperative courses and complications.

Upon admission, all enrolled patients were routinely subjected to laboratory tests. Medical examinations included US, abdominal computed tomography (CT, plain scan or contrast-enhanced scan), magnetic resonance cholangiopancreatography (MRCP), upper gastrointestinal imaging and gastroscopy/colonoscopy were performed based on the patients' conditions. Patients combined with choledocholithiasis were subjected to an endoscopic retrograde cholangiopancreatography (ERCP) procedure selectively before cholecystectomy. Notably, 21 of the 29 patients accepted laparoscopic surgery with a standard four-port technique, and 8 conversion cases were found due to dense adhesion between gallbladder and adjacent gastrointestinal tract. The following key considerations were taken into account during surgical management: (a) For CEF patients without GI, cholecystectomy + Fistula repair and cholecystectomy + choledocholithotomy + T-tube insertion+ Fistula repair could be selected based on the patients' conditions; (b) Fistula repair was accomplished by way of simple interrupted sutures, applying Endo-GIA device or Billroth II (complete resection of fistula) for the CEF patients with and without GI; and (c) For the CEF patients with GI, surgical management included simple enterolithotomy, enterolithotomy + cholecystectomy + closure of cholecystoenteric fistula (one-stage procedure) or enterolithotomy + cholecystostomy. Surgical relief of bowel obstruction remains the mainstream of GI.

Statistical analyses were performed using an Excel software 15.30 (Microsoft Corporation, Redmond, WA, USA). Continuous variables were expressed as medians (range), whereas categorical ones were presented as frequencies (percentages).

## Results

A total of 29 patients were included in our study over the 11-year period. Among them, 14 (48.3%) and 15 (51.7%) patients were male and female, respectively, with a median age 66 years (range: 35–95 years) (Table 1). Analysis of subtype distribution of CEF indicated that seventeen (58.6%) patients had CDF, six (20.7%) had CCF, three (10.3%) exhibited CGF, one (3.4%) patient presented with both CDF and CGF while two (6.8%) patients presented both Mirizzi syndrome and CDF (Table 3). With regards to other conditions, eight (27.6%) patients had diabetes mellitus (DM), thirteen (44.8%) had hypertension, three (10.3%) had cardiac and coronary disease, four (13.8%) had chronic pulmonary disease while seven (24.1%) exhibited gastric or duodenum ulcers. Thirteen (44.8%) patients presented with >5-year medical history of cholelithiasis. Notably, abdominal pain, especially the right upper quadrant abdominal pain, was the main complain, accounting for 75.9% of all the patients, followed by nausea (44.8%), vomiting (27.6%) and fever (20.7%). Decreased passing gas and defecation was observed in thirteen (44.8%) patients, which was typical presentation of bowel obstruction. Fluctuations among laboratory indices included elevated white blood cells (six patients, 20.7%), elevated levels of C-reaction protein (eighteen patients, 62.1%), increased levels of total bilirubin (six patients, 20.7%) and elevated levels of alanine aminotransferase and aspartate aminotransferase (two patients, 6.9%) (Table 1). Of all the 29 patients, seventeen (58.6%) patients achieved a preoperative diagnosis while the remaining 12 (41.4%) were diagnosed during the operation (Table 3).

**Table 1:** Basic clinical dates of CEF with and without GI.

Clinical findings (*n* = 29)	*n*	%
Sex		
Male	14	48.3
Female	15	51.7
Age(years)		
≤60	10	34.5
60–80	12	41.4
≥80	7	24.1
BMI (kg/m^2^)		
≤18.5	2	6.9
18.5–24	21	72.4
≥24	6	20.7
Coincidental disease		
Diabetes mellitus	8	27.6
Hypertension	13	44.8
Cardiac & coronary disease	3	10.3
Chronic pulmonary disease	4	13.8
Gastric or duodenum ulcer	7	24.1
Course of cholelithiasis(years)		
≤5	16	55.2
5–10	2	6.9
≥10	11	37.9
Clinical manifestation		
Abdominal pain	22	75.9
Nausea	13	44.8
Vomiting	8	27.6
Fever (≥37.5°C)	6	20.7
Decreased passing gas and defecation	13	44.8
Laboratory test		
Elevated of WBC count	6	20.7
Elevated of CRP	18	62.1
Elevated of TBIL	6	20.7
Elevated of ALT & AST	2	6.9

*WBC, white blood cell; CRP, C-reaction protein; TBIL, total bilirubin; ALT, alanine aminotransferase; AST, aspartate aminotransferase.*

**Table 2:** Appearance of medical examinations in all the patients enrolled in this study.

Medical examinations	Appearance	*n* (%)
US (*n* = 24)	Thick-walled gallbladder	14(58.3)
	Gallbladder atrophy	12(50)
	Pneumobilia	5(20.8)
	Pneumo-gallbladder or air-fluid level in gallbladder	2(8.3)
	Fulfilled gallstone	12(50)
	Disappear of gallbladder	4(16.7)
CT (*n* = 29)	ill-defined borderline between gallbladder and digestive tract	18(62.1)
	Thick-walled gallbladder	20(69)
	Pneumobilia	18(62.1)
	Pneumo-gallbladder or air-fluid level in gallbladder	11(37.9)
	Bowel loops dilatation	12(41.4)
MRCP (*n* = 17)	ill-defined borderline between gallbladder and digestive tract	7(41.2)
	Thick-walled gallbladder	13(76.5)
	Gallbladder atrophy	10(58.8)
	Pneumobilia	10(58.8)
	Pneumo-gallbladder or air-fluid level in gallbladder	5(29.4)
Upper gastrointestinal Imaging (*n* = 2)	Observation of communication between gastrointestinal and gallbladder	1(50)
Gastroscopy (*n* = 7) Colonoscopy (*n* = 1)	Observation of fistula	4(57.1)
	Observation of fistula	0(0)

**Table 3:** Intraoperation findings of CEF with and without GI.

intraoperation findings (*n* = 29)	*n* (%)	value
Diagnostic characteristics for CEF		
Preoperative considerations	17(58.6)	–
Intraoperative diagnosis	12(41.4)	–
Distribution of CEF		
CDF without GI/CDF with GI	6(20.7)/11(37.9)	–
CCF without GI /CCF with GI	6(20.7)/0(0)	–
CGF without GI /CGF with GI	2(6.9)/1(3.4)	–
CDF without GI and CGF without GI	1(3.4)	–
Mirizzi syndrome and CDF	2(6.8)	–
Surgical method		
Laparoscopy	13(44.8)	–
Conversion	8(27.6)	–
Open	8(27.6)	–
Surgical procedure		
^a^Enterolithotomy	6(20.7)	–
^a^Enterolithotomy + cholecystectomy + fistula repair	5(17.2)	–
^a^Enterolithotomy + cholecystostomy	1(3.4)	–
^b^Cholecystectomy + fistula repair	11(37.9)	–
^b^Cholecystectomy + choledocholithotmy + fistula repair + T-tube insertion	5(17.2)	–
^b^Cholecystectomy + Billroth II	1(3.4)	–
Closure method of the fistula (*n* = 20)		–
Fistula resected and simply interrupted sutures	18(90)	–
Applyingby Endo-GIA device	1(5)	–
Billroth II (completely fistula resection)	1(5)	–
Histopathologic revealing gallbladder cancer (*n* = 22)	1(4.5)	–
Operation time (min)	–	157(65–360)
Blood loss (ml)	–	40(10–450)
Size of fistula (cm)	–	1.4(0.5–3)
Postoperative ICU management	4(13.8)	–
Hospital stays (days)	–	16(5–40)

^*a*^ *CEF with GI.*

^*b*^ *CEF without GI.*

**Figure 1:**
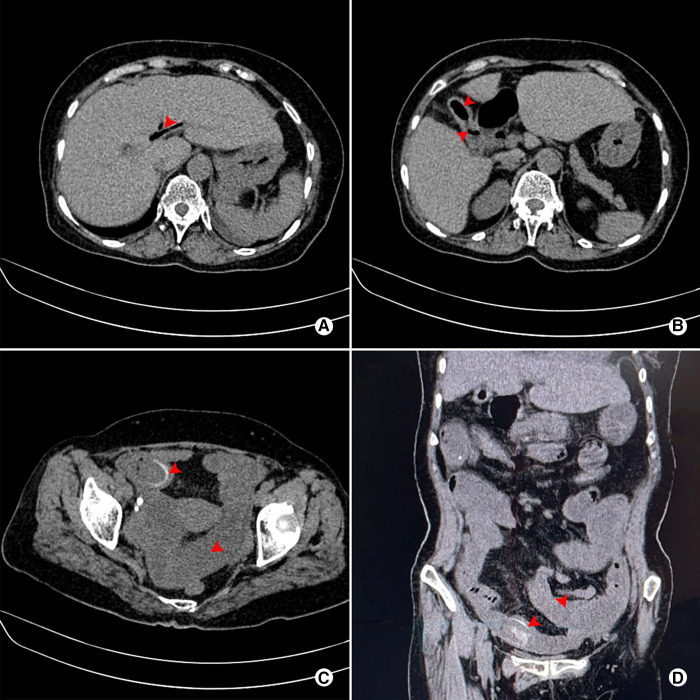
Abdominal plain CT scan of a 71-year-old female patient who was preoperatively diagnosed with CDF combined with GI. **(A)** Pneumobilia (red arrow); **(B)** Pneumo-gallbladder and ill-defined borderline between gallbladder and digestive tract (red arrow); **(C)** Rim-calcified gallstone impacted in the small bowel, combined with dilatation of proximal small bowel (red arrow); **(D)** Coronal view of impacted rim-calcified gallstone and dilatation of proximal small bowel (red arrow).

**Figure 2:**
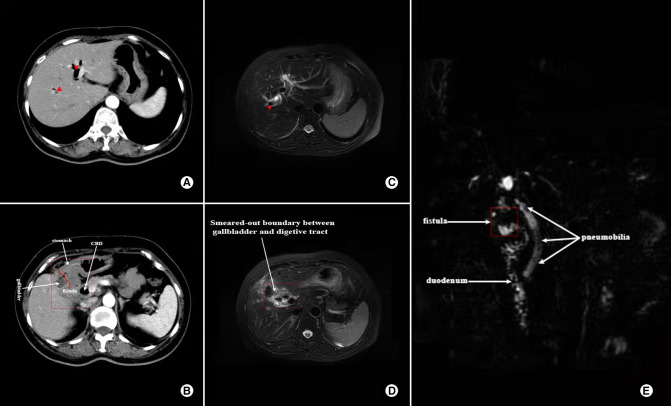
Contrast-enhanced CT and MRCP images showing the characteristics of a 57-year-old female who was diagnosed with both CDF and CGF; **(A)** CT image of Pneumobilia (red arrow); **(B)** CT image showing ill-defined borderline between gallbladder and digestive tract (marked in the red box); **(C)** MRCP image showing Pneumobilia (red arrow); **(D)** MRCP image of ill-defined borderline between gallbladder and digestive tract (marked in the red box); **(E)** Reconstruction of MRCP showing Pneumobilia and fistula (white arrow).

As a convenient and cost-effective examination, US was applied to twenty-four patients as a routine test. Consequently, US provide significant clues including thick-walled gallbladder (58.3%), Gallbladder atrophy (50%), Pneumobilia (20.8%) and Pneumo-gallbladder or air-fluid level in gallbladder (8.3%). All the 29 patients accepted abdominal CT, either plain scan or contrast-enhanced scan. Typical signs observed in the abdominal CT included ill-defined borderline between gallbladder and digestive tract, thick-walled gallbladder, pneumobilia, pneumo-gallbladder or air-fluid level in gallbladder, as well as bowel loop dilatation (Figures 1A–D, 2A,B). MRCP was applied to seventeen patients. Notably, similar signs to those observed in abdominal CT were evident, except bowel loops dilatation (Figures 2C–E). Two patients accepted upper gastrointestinal imaging, one of whom was diagnosed CDF by observation communication between gastrointestinal and gallbladder. Moreover, seven patients accepted gastroscopy, three were diagnosed with CDF while one was diagnosed with both CDF and CGF through observation of fistula (Table 2).

**Figure 3:**
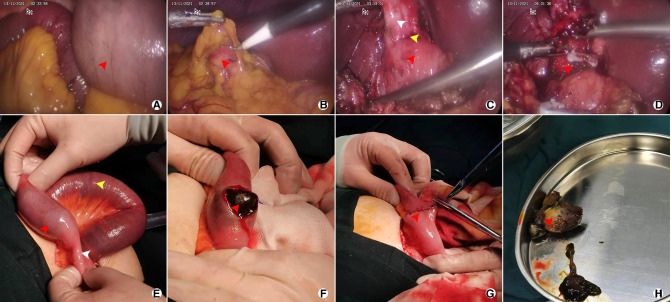
Representative images after operation of the 71-year-old female patient who was diagnosed CDF with GI; **(A)** Dilatation of small bowel (red arrow); **(B)** Atrophic gallbladder covered by great omentum; **(C)** CDF from a laparoscopic view, including atrophic gallbladder (white arrow), ill-defined borderline (yellow arrow), and duodenum (red arrow); **(D)** Partial cholecystectomy and fistula (red arrow); **(E)** Impacted gallstone (red arrow), dilatation of small bowel (yellow arrow) and emptiness of distal intestinal (white arrow); **(F)** Enterolithotomy (longitudinal incision, red arrow); **(G)** Closure of the intestinal (red arrow); **(H)** gallstone extracted from small bowel (red arrow).

Among the seventeen patients without GI, eight had CDF, six CCF, two exhibited CGF, while one patient had both CDF and CGF. Laparoscopy procedure was performed on sixteen patients. Among them, ten successfully underwent laparoscopy procedure while the other six underwent conversion to open surgery due to dense adhesion around the Calot's triangle, bleeding and intolerant operation time. Total or subtotal cholecystectomy was performed in all the seventeen patients and five patients underwent choledocholithotmy and T-tube insertion due to choledocholithiasis. One patient had both CDF and CGF, with fistula sizes of 2 and 1 cm, respectively. The Billroth II procedure was performed for the exceptional case (Table 3).

Eleven of the twelve patients with GI were diagnosed with CDF, with the remaining one exhibiting CGF. A majority of the cases were accomplished in an emergency manner (9 patients, 75%). Furthermore, laparoscopy procedure was tried in five patients with the remaining seven subjected to open surgery. Consequently, three and two patients successfully underwent laparoscopy procedure and conversion to open surgery, respectively. Seven patients underwent simple Enterolithotomy. Four patients underwent a one stage procedure which indicated finishing Enterolithotomy, cholecystectomy and closure of cholecystoenteric fistula. Distribution of location of impacted gallstone were as follows: jejunum (6 patients, 50%), ileum (4 patients, 33.3%) and ileocecal (2 patients, 16.7%). The median maximum diameter of the impacted gallstone was 3.8 cm (range: 2–6 cm) (Table 4).

**Table 4:** Details for all patients diagnosed with CEF combined with GI.

Number	Gender Age(years)	ASA score	Surgical time	Type of CEF	Size of impacted gallstone(cm)	Location of impacted gallstone	Surgical procedure	Length of operation (min)	Blood loss(ml)	Hospital Stays (days)	prognosis
1	M/68	II	Elective	CDF	3*3	Ileum	Conversion + one stage procedure	190	20	40	Wound infection
2	F/91	III	Emergency	CDF	6*3	Jejunum	Laparotomy + Enterolithotomy	110	10	8	Uncomplicated
3	F/88	IV	Emergency	CDF	3*3	Jejunum	Enterolithotomy	110	70	28	Wound infection; Pulmonary infection
4	M/89	III	Emergency	CDF	2*1	Jejunum	Enterolithotomy	65	10	12	Uncomplicated
5	M/67	II	Elective	CGF	4*3	Jejunum	Conversion + one stage procedure	165	40	14	Renal insufficiency
6	F/87	IV	Emergency	CDF	3.5*3	Jejunum	Enterolithotomy +	270	100	12	Cardiac insufficiency
7	M/60	II	Emergency	CDF	5*4	Ileum	Laparotomy + Enterolithotomy	105	10	9	Uncomplicated
8	F/96	III	Elective	CDF	4*3	Ileocecal valve	Enterolithotomy	65	10	18	Uncomplicated
9	F/66	II	Emergency	CDF	3*3	Ileocecal valve	Enterolithotomy + cholecystostomy	85	20	7	Uncomplicated
10	F/82	III	Emergency	CDF	3*3	Jejunum	one stage procedure	155	30	30	Wound infection; fistula recurrence
11	F/71	III	Emergency	CDF	4*3,2*1	Ileum	Laparotomy + one stage procedure	200	80	16	Uncomplicated
12	M/62	III	Emergency	CDF	5*4	Ileum	Enterolithotomy	74	20	18	Uncomplicated

*M, male; F, female; One stage procedure, enterolithotomy + cholecystectomy + closure of cholecystoenteric fistula.*

A total of twenty patients underwent primary closure of fistula, by means of simple interrupted sutures (18 patients, 90%), application of the Endo-GIA device (1 patient, 5%) and Billroth II procedure (1 patient, 5%). The median operation time and blood loss were 157 min (range: 65–360 min) and 59 ml (range: 10–450 ml), respectively. Four patients (13.8%) were transferred to the intensive care unit due to unstable hemodynamics or sepsis secondary to GI or cholangitis. The median length of hospital stay was 17 days (5–40). Post-operative histopathologic finding revealed gallbladder cancer in one patient.

Overall, the post-operative complication rate was 36.4%, including wound infection in four patients (18.2%), failure of fistula repair in three patients (13.6%) and residual stone in common bile duct in one patient (3.4%). Most of the complications required additional surgery, including negative pressure drainage for wound infection, and ERCP combined with sphincterotomy for residual stone in common bile duct. With regards to fistula recurrence, two patients (CCF) received ultrasound-guided catheter drainage while one patient (CGF) recovered after conservative treatment. No deaths were reported in our study (Table 5).

**Table 5:** Surgical complications of CEF with and without GI.

Surgical complications (*n* = 29)	*n* (%)
Failure of fistula repair (*n* = 22)	3(13.6)
Wound infection	4(13.8)
Residual stone in common bile duct	1(3.4)
Mortality	0(0)

## Discussion

**Figure 4:**
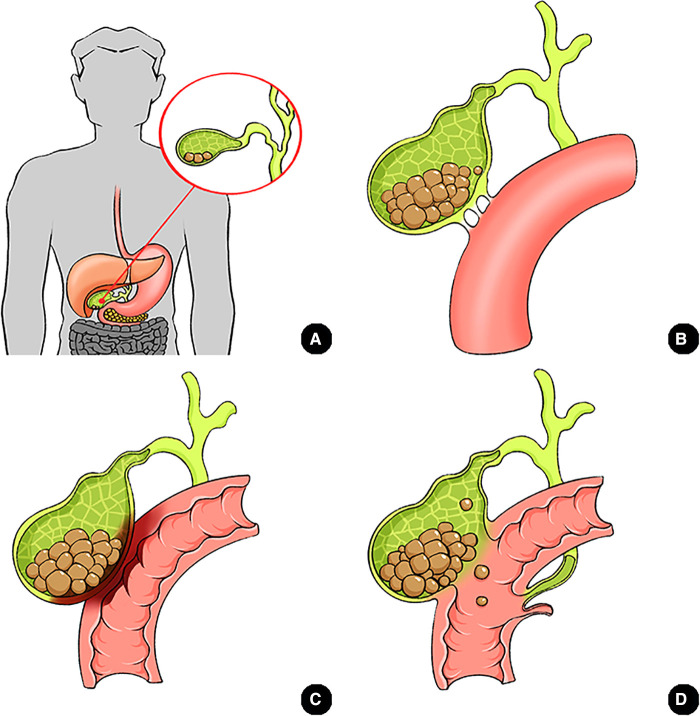
Schematic diagram of the formation of CEF with GI. **(A)** gallstones; **(B)** Dense adhesion between gallbladder and duodenum; **(C)** Compression of fulfilled gallstones cause tissue ischemia, necrosis and erosion; **(D)** Fistula formation and gallstones migrate into digestive tract causing gallstone ileus.

CEF, a condition first described by Thomas Bartholin in 1,654 and which allows gallstones to migrate into the digestive tract, is a rare complication of gallstone disease (7). The condition tends to predominantly occur in the female geriatric population which is partially confirmed in the present study (19/29 patients, 65.5%, over 60 years; 15/29 patients, 51.8%, female). These dates are slightly below international literature reported (80% female) (2, 8, 9). CEF most frequently presents in the duodenum (75%–80%), followed by colon and stomach. The detail dates recorded in the present study were 58.6% in CDF, 20.7% in CCF, 10.3% in CGF, respectively which were consistent with those reported in previous studies (4, 5, 9). The mechanism underlying of CEF is currently clear. Briefly, large gallstones (especially fulfilled gallstones) compress the gallbladder wall thereby causing wall ischemia, necrosis, erosion and eventually fistula formation with the adjacent viscus (Figure 4) (3, 9). According to Gonzalez-Urquijo et al. (9) and Glenn et al. (10), frequent acute cholecystitis and biliary calculus impaction cause dense adhesion, which ultimately leads to erosion between the gallbladder and the adjacent viscus. These consequently lead to fistula development.

Gallstones may migrate into the digestive tract (stomach, duodenum or colon), thereby resulting in bowel obstruction, a condition known as gallstone ileus. This condition most commonly affects the terminal ileum or ileocecal valve, and is less common in the jejunum, colon and stomach (Bouveret's syndrome) (1, 11–14). Most of the impacted gallstones are bigger than 2.5 cm in size (Figure 3F,H), normally ranging from 2 to 5 cm (1). Results from the present study indicated that all the impaction site of GI occurred in small intestinal, including jejunum (6 patients, 50%), ileum (4 patients, 33.3%) and ileocecal (2 patients, 16.7%), with a median size of 3.8 cm (range: 2–6 cm). The minute difference of the impaction site may be attributed to the large size of the gallstones that couldn't pass through the jejunum.

Clinical presentation of CEF is atypical and indistinguishable relative to uncomplicated cholelithiasis (2, 9). Results from the present study indicated that most patients (26/29) had a long history of cholelithiasis. Notably, the major symptoms in CEF patients included abdominal pain (common in right upper quadrant), nausea and vomiting. Additionally, decreased passing gas and defecation was quite common among CEF patients with GI.

Despite major advancement in imaging techniques and gastrointestinal endoscopy, preoperative diagnosis of a CEF remains challenge. And the accurate preoperative diagnosis is of great importance for surgeons to avoid unanticipated complex, prolonged and headache procedure. Li et al. (4) and Gonzalez-Urquijo et al. (9) reported that successful preoperative diagnosis of CEF in 31% and 40%, respectively. In the present study, we found a preoperative diagnosis of 58.6% (17/22), which was slightly higher than that reported in previous literatures mentioned above. The experience of our institution regarding improvement of preoperative diagnosis is as follows: (1) Long time (especially > 5 years) of repeated episodes of cholecystitis is a risk factor, thus should capture surgeons' attention; (2) Although accurate diagnosis by US detection can be difficult, signs such as thick-walled gallbladder, gallbladder atrophy and pneumobilia/pneumo-gallbladder are valuable clues of CEF; (3) Abdominal CT (plain or contrast-enhanced scan), especially coronal reconstruction, is imperative to effective identification with signs of ill-defined borderline between gallbladder and digestive tract. Moreover, when combined with thick-walled gallbladder, Pneumobilia, pneumo-gallbladder or air-fluid level in gallbladder, the surgeon should highly suspect the presence of CEF; (4) MRCP has similar signs to CT scan. Notably, the MRCP is much more helpful when a CEF is combined with choledocholithiasis or Mirizzi syndrome; (5) Upper gastrointestinal imaging, gastroscopy/colonoscopy should be considered when a CEF is suspected, which can observe the fistula or communication between gastrointestinal and gallbladder; and (6) For patients who present with intestinal obstruction, typical signs which is also known as Rigler's triad (15–17), including bowel loop dilatation, impaction of rim-calcified or total-calcified gallstone associating with the above signs, make the diagnosis relatively easy.

Therapeutic principle for patients with CEF and CEF with GI is vastly different. Notably, cholecystectomy and closure of fistula is advocated, for CEF patients, either by an open or a laparoscopic procedure based on the surgeons' experience and patients' condition. (2–4, 9) Traditionally, most scholars advise CEF should be managed in an open manner because of the dense adhesion and the obscured Calot's triangle (4, 18, 19). Fortunately, numerous studies have shown that laparoscopic surgery is not a contraindication for CEF, owing to the recent advancement of technology coupled with widespread application of laparoscopy (2, 9, 10). Furthermore, Vanessa et al. (20) described a case report regarding CEF managed by robotic surgery. However, other studies have shown that the rate of conversion to open can be very high due to heavy inflammation, bleeding and difficulty in intestinal suturing (4, 21, 22). In this study, thirteen patients (44.8%) successfully accepted laparoscopic procedure, while eight (27.6%) underwent conversion to open procedure. The method of closing the sinus tract mainly involves sutures, applying Endo-GIA device and Graham patch (4, 9, 23). In the present study, majority sinus tracts (90%) were closed by simple interrupted sutures. One special case was a 57-year-old woman who both presented with CDF (fistula size: 2 cm) and CGF (fistula size: 1 cm). Even worse, the two fistulas were so close which made the simply interrupted sutures or Graham patch repair too difficult and insecure. Finally, a Billroth II (completely fistula resection) was performed, and the patient discharged without complication on post-operation day 18 (POD 18).

For CEF patients with GI, surgical treatment approach has generated a sustained debate on whether the biliary surgery should be carried out in the meantime to relieve bowel obstruction (11, 24). Current surgical procedures are summarized as follows: (1) enterolithotomy (2) enterolithotomy, cholecystectomy, and fistula repair (one-stage procedure) and (3) enterolithotomy with cholecystertomy + fistula repair later (two-stage procedure) (1). To date, the literature on CEF with GI is composed mainly of cases series or case report and whether the fistula should be repaired or not remains unanswered. Moreover, none of the studies are randomized control trial and the selections of the surgical strategy are likely to be influenced by the surgical bias (1, 14). Simple enterolithotomy is associated with lower operative morbidity and mortality, especially for the aged patients or patients with concomitant disease (1, 24, 25). Clavien et al. (24) suggests that one stage procedure is safe and worth promoting when the local and general conditions permit. Two stage procedure, in fact, is not common and the delayed cholecystectomy and fistula repair is suitable in case of symptoms persistence (1, 25, 26). The results from the present study showed that seven (58.3%) and 4 patients (33.3%) accepted simple enterolithotomy and one-stage procedure, respectively, indicating that enterolithotomy is the most commonly preferred procedure.

In the present study, failure of fistula repair was the most serious complication, which eventually resulted in intraperitoneal infection. In total, three patients presented with fistula recurrence, of which two and one had CCF on POD 6 and CDF on POD 5, respectively. Two patients were successfully treated through catheter drainage, while one (CDF) recovered after conservative treatment. One patient presented with jaundice, abdominal pain and bile leakage and was confirmed a residual stone in CBD on POD 3 and the ERCP and sphincterotomy was performed. We observed no death case in this study, which was markedly less than that reported 6.7%–25% in the literature (9, 27).

In conclusion, CEF is a rare complication of gallstone disease, that is occasionally found during operation. High grade suspicion is indispensable for the diagnosis of CEF. Moreover, a combination of US, CT, MRCP, gastrointestinal imaging and gastroscopy/colonoscopy could make the diagnosis more accurate. Surgical management of CEF, regardless of whether it is laparoscopy or open, is essential and should be performed by experienced surgeons. At present, there is no consensus on the best surgical approach of CEF, and the main difficulty is to successfully repair the fistula. For CEF patients with GI, the primary goal is to relieve the intestinal obstruction and fistula closure should be avoided unless the patients' conditions are good enough.

## Data Availability

The raw data supporting the conclusions of this article will be made available by the authors, without undue reservation.
